# Impact of congenital anomalies on infant mortality in the 14^th^ Health Region of Paraná, from 2010 to 2019

**DOI:** 10.1590/1984-0462/2026/44/2025182

**Published:** 2026-03-23

**Authors:** Nitza Ferreira Muniz, Ayoade Desmond Babalola, Maria Antonia Ramos Costa, Lavinia Schuler-Faccini

**Affiliations:** a Universidade Federal do Rio Grande do Sul, Porto Alegre, RS, Brazil.; b Universidade Estadual do Paraná, Paranavaí, PR, Brazil.

**Keywords:** Congenital abnormalities, Underlying cause of death, Infant mortality, Health information systems, Public health surveillance, Anormalidades congênitas, Causa básica de morte, Mortalidade infantil, Sistemas de informação em saúde, Vigilância em saúde pública

## Abstract

**Objective:**

The aim of this study was to describe congenital anomaly (CA) notifications at birth, evaluating their impact on infant mortality in northwest Paraná.

**Methods:**

This is a cross-sectional study using linked data on births with CA and deaths due to these conditions in the 14^th^ Health Region of Paraná, Brazil (2010–2019), obtained through the linkage of data from the Live Birth Information System (Sinasc) and the Mortality Information System (SIM).

**Results:**

A total of 216 live births (LBs) with CAs were identified in Sinasc (57.7/10,000 LBs), of which 71.8% were isolated anomalies and 28.2% were multiple anomalies, totaling 331 reported anomalies. Limb and musculoskeletal defects were the most frequent (25.7%). After linking Sinasc and SIM, congenital heart defects (CHDs), initially ranked seventh, became the second most prevalent CA. A total of 115 infant deaths due to CA were identified, resulting in an infant mortality rate (IMR) of 30.7/10,000 LBs, with CHDs and neural tube defects (NTDs) as the leading causes. The data linkage revealed 55 deaths with CAs not recorded in Sinasc, increasing prevalence to 72.4/10,000 LBs (+25.5%).

**Conclusions:**

IMR for CAs in the region exceeded the national average (28.0/10,000 LBs), highlighting the importance of linking data to strengthen epidemiological surveillance of these conditions. CHDs and NTDs were the most frequent causes of infant deaths associated with CAs, reinforcing the need to improve early detection and the recording of these cases to reduce infant mortality due to CAs across different regions of Brazil.

## RESUMO

**Objetivo:**

Descrever as notificações de Anomalias Congênitas (AC) ao nascer e avaliar seu impacto na mortalidade infantil no noroeste do Paraná.

**Métodos:**

Estudo transversal com dados vinculados de nascimentos com AC e óbitos por essas condições, na 14ª Regional de Saúde do Paraná, Brasil (2010-2019), obtidos por meio do relacionamento dos dados do Sistema de Informações sobre Nascidos Vivos (Sinasc) e do Sistema de Informações sobre Mortalidade (SIM).

**Resultados:**

Foram identificados 216 Nascidos Vivos (NV) com AC registradas no Sinasc (57,7/10 mil NV), sendo 71,8% isoladas e 28,2% múltiplas, totalizando 331 anomalias. Defeitos de membros e osteomusculares foram os mais frequentes (25,7%). Após a vinculação Sinasc-SIM, as cardiopatias congênitas, inicialmente na sétima posição, passaram à segunda entre as AC mais prevalentes. Foram identificados 115 óbitos infantis por AC, resultando em uma Taxa de Mortalidade Infantil (TMI) de 30,7/10 mil NV, com cardiopatias e Defeitos do Tubo Neural (DTN) como as principais causas. A vinculação revelou 55 óbitos com AC não registrados no Sinasc, elevando a prevalência para 72,4/10 mil NV (+25,5%).

**Conclusões:**

A TMI por AC na região superou a nacional (28/10 mil NV), evidenciando a importância da vinculação de dados para fortalecer a vigilância epidemiológica dessas condições. As cardiopatias congênitas e os DTN foram as causas de óbito infantil mais frequentes associadas às AC, reforçando a necessidade de aprimorar a detecção precoce e o registro desses casos a fim de reduzir a mortalidade infantil por AC em diferentes regiões do Brasil.

## INTRODUCTION

The term “Congenital Anomalies” (CAs) refers to any structural (physical) or functional (metabolic, for example) abnormality that occurs during intrauterine development and is present at birth, whether detected immediately or later in life. Many can be detected already during the prenatal period.^1^ Its etiology involves genetic (gene or chromosomal disorders), environmental (teratogens), or, most frequently, multifactorial (where multiple factors, such as nutrition and environmental exposures, interact with the individual’s genetic predisposition).^2^

Globally, between 3% and 6% of live births (LBs) have some type of CA, contributing significantly to the global burden of disease. More than 90% occur in low- and middle-income countries.^3^ In Brazil, it is estimated that approximately 24,000 births with some type of CA occur each year; however, this estimate is likely to be underestimated.^4^

Every year, CAs are responsible for more than 240,000 deaths in the neonatal period worldwide.^1^ In Latin America, CAs are between the second and fifth leading causes of death in children under 1 year of age.^5^ In Brazil, CA advanced from the fifth to the second leading cause of infant death from 1990 to 2020, due to the overall reduction in infant mortality due to acute causes.^6,7^

Since 2010, the state of Paraná has shown a decline in the reported prevalence of CA,^8^ possibly reflecting improvements in prenatal care and health services. However, CA remained the second leading cause of infant mortality throughout the 2010–2019 period,^8^ and concerns about underreporting persist. A time series study identified the western region of Paraná as one of the areas with the highest positive relative risk for CAs in Brazil.^9^

Similarly, in the 14^th^ Health Region, CAs remained the second leading cause of infant mortality, despite a reported decline in prevalence during the same period.^8^ Despite their recognized impact, there are still gaps in understanding the actual burden of CAs at the regional level, largely due to limitations in routine data recording. In this context, this study aims to describe the registration of LBs with CAs and assess their impact on infant mortality in northwestern Paraná (2010–2019).

## METHOD

This is a cross-sectional study, based on data linked to births and deaths in the 14^th^ Health Region of Paraná, covering the period from 2010 to 2019. The Paraná State is divided into 22 Health Regions within four macro-regions.^10^ The 14^th^ Region, part of the northwest macro-region, is headquartered in Paranavaí and comprises 28 municipalities. It covers an area of 9,833.663 km^2^, with an estimated population of 272,818 and a Human Development Index of 0.706. Paranavaí has the largest population (92,001), while Nova Aliança do Ivaí has the smallest (1323).^11^

LBs were identified in the Live Birth Information System (Sinasc) database, while deaths were identified in the Mortality Information System (SIM) database, through the Department of Informatics of the Unified Health System,^8^ and were collected between July 2019 and June 2020. Sinasc is fed by an official document, the Declaration of Live Births, which contains information on the mother’s gestational history and the newborn. Since 2001, the Declaration of Live Births has included a specific field for recording the codes for CAs observed at birth,^12^ as described in Chapter XVII (Q00-Q99) of the 10th Revision of the International Classification of Diseases (ICD-10).^13^

SIM database, in turn, is fed by the Death Certificate, which contains information about the parents, the deceased, and the cause of death, including CA-related causes coded according to ICD-10.^14^ After standardizing these two databases (standardizing the main variables), linkage was performed to create a single database using Microsoft^®^ Office Excel. The first stage, deterministic in nature, was conducted by identifying the unifying variable common to both systems, the Declaration of Live Births number. Subsequently, a manual comparison was carried out for the cases that remained unmatched in the first stage, using the variables available in both databases: mother’s name, municipality of residence, newborn’s name, biological sex, and date of birth.

Records of births with CAs as well as infant deaths with CAs as the cause found in Sinasc and SIM were included. These anomalies were grouped according to Chapter XVII of the ICD-10^13^ and the Ministry of Health’s list of priority anomalies for surveillance.^15^ Only children who were born and died between 2010 and 2019 were included in the study, considering only those who died with less than 365 days of life. Thus, deaths of children under 1 year of age that occurred in 2020 but were born in 2019 were missed, as well as infant deaths in 2010 of LBs from the previous year. This issue is due to the nature of the data, which are cross-sectional rather than longitudinal (birth cohorts). In addition, we also included and analyzed CA deaths that occurred in the period, but no records were found in Sinasc.

The overall prevalence of CAs was calculated as the number of LBs with CAs (field 6 of the Live Birth Certificate marked “yes” and field 41 filled with the ICD-10 code), divided by the total number of LBs and multiplied by 10,000. Only one case had field 6 marked without completion of field 41 and was therefore excluded from the analysis. In addition, infant deaths (up to 1 year of age) with a description of CAs in SIM were considered to estimate the potential increase in prevalence from deaths due to CAs that had not been recorded in Sinasc. For the IMR due to CAs, the SIM records were linked to Sinasc, and the ratio between the total number of CA deaths recorded in the SIM in children under 1 year of age and the total number of LBs in that period, multiplied by 10,000, was calculated.

This article is part of a larger project, which was approved by the Research Ethics Committee Involving Human Beings of the State University of Maringá (UEM) (Opinion No. 3.211.733/20 of March 2019) and by the Research Ethics Committee of the Workers’ Hospital of the Paraná State Health Department (SESA-PR) (Opinion No. 3.440.478/05 of July 2019).

## RESULTS

Between 2010 and 2019, 37,428 LBs were registered from mothers living in the 14^th^ Health Region of Paraná; 216 (57.7/10,000) of them had some CAs recorded in Sinasc. Of these, 155 (71.8%) were classified as isolated anomalies, and 61 (28.2%) had multiple anomalies. In the same period, 376 deaths of children under 1 year of age were reported, of which 115 (30.6%) had some CAs recorded as the cause of death, resulting in an IMR per CA of 30.7/10,000 LBs. After linking Sinasc and SIM, it was found that 55 deaths due to CAs had not had the anomaly recorded in Sinasc. Thus, the number of LBs with CAs increased from 216 to 271, raising the prevalence of LBs with CAs to 72.4/10,000 LBs, an increase of 25.5%.

**Figure 1.**
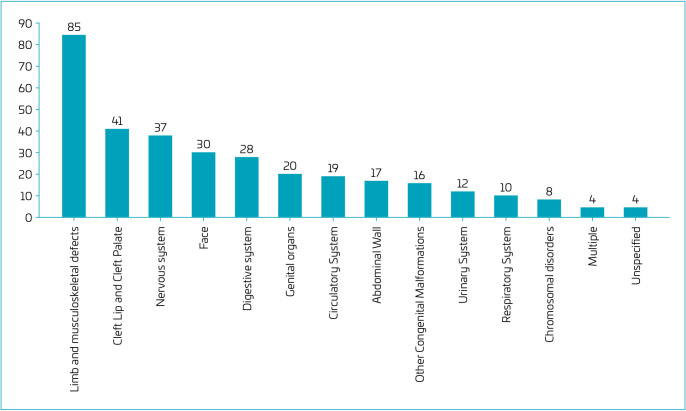
Number of congenital anomalies recorded in the Declaration of Live Births (Sinasc) in the 14^th^ Health Region of Paraná between 2010 and 2019.

Table 1 and Figure 1 show the CAs recorded at birth in Sinasc. The most frequent were those visible at birth, such as limb and musculoskeletal defects (25.7%), with a predominance of foot deformities and polydactyly, followed by oral clefts (12.4%) and neural tube defects (NTDs) (11.2%), especially anencephaly and spina bifida.

**Table 1.** Anomalies recorded in the Declaration of Live Births (Sinasc) in the 14^th^ Health Region of Paraná between 2010 and 2019.

Congenital anomalies	n (%)	Prevalence (10,000)
Limb and musculoskeletal defects	85 (25.7)	22.7
Cleft lip and cleft palate	41 (12.4)	11.0
Nervous system	37 (11.2)	9.9
Face	30 (9.1)	8.0
Digestive system	28 (8.5)	7.5
Genital organs	20 (6.0)	5.3
Circulatory system	19 (5.7)	5.1
Abdominal wall	17 (5.1)	4.5
Other congenital malformations	16 (4.8)	4.3
Urinary system	12 (3.6)	3.2
Respiratory system	10 (3.0)	2.7
Chromosomal disorders	8 (2.4)	2.1
Multiple	4 (1.2)	1.1
Unspecified	4 (1.2)	1.1
Total CAs	331 (100)	-
Total number of individuals with recorded CAs*	216 (100)	57.7

*The total number of anomalies is more than 216 since some children have more than one anomaly. CAs: Congenital anomalies.

**Figure 2.**
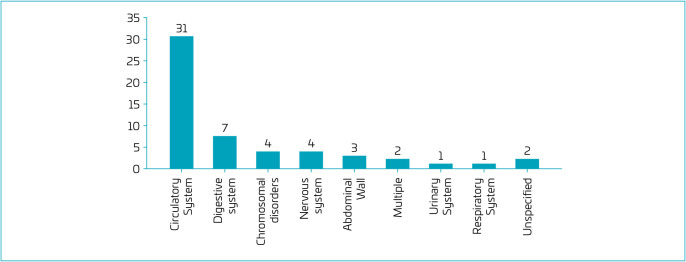
Number of congenital anomalies in death certificates (SIM) and without registry in the Declaration of Live Births (Sinasc) in the 14^th^ Health Region of Paraná between 2010 and 2019.

Table 2 and Figure 2 show the CAs reported in SIM that were not recorded in Sinasc. Congenital heart defects (CHDs) stand out, accounting for the majority of cases (56.4%). However, more than half of these (61.3%) were not specified as to type. In addition, some anomalies that were easily detectable at birth, such as anencephaly (n=2) and gastroschisis (n=2), were also not recorded in the Sinasc.

**Table 2.** Anomalies in death certificates (SIM) and without registry in the Declaration of Live Births (Sinasc).

Congenital anomalies (ICD-10)	n (%)
Circulatory system	31 (56.4)
Unspecified (Q248, Q249)	19 (34.5)
Hypoplastic heart syndrome (Q234)	5 (9.1)
Ventricular–atrial communication (Q203)	2 (3.6)
Ebstein’s anomaly (Q225)	1 (1.8)
Others (Q204, Q208, Q240, Q246)	4 (7.3)
Digestive system	7 (12.7)
Esophageal atresia (Q390, Q391)	2 (3.6)
Intestinal absence and atresia (Q418, Q419)	2 (3.6)
Diaphragmatic hernia (Q790)	1 (1.8)
Hypertrophic pyloric stenosis (Q400)	1 (1.8)
Unspecified (Q439)	1 (1.8)
Chromosomal disorders	4 (7.3)
Edwards syndrome (Q913)	2 (3.6)
Down syndrome (Q909)	1 (1.8)
Patau syndrome (Q917)	1 (1.8)
Nervous system	4 (7.3)
Anencephaly (Q000)	2 (3.6)
Hydrocephalus (Q039)	2 (3.6)
Abdominal wall	3 (5.5)
Gastroschisis (Q793)	2(3.6)
Prune-Belly (Q794)	1(1.8)
Multiple (Q897)	2 (3.6)
Urinary system (Q602)	1 (1.8)
Respiratory system (Q336)	1 (1.8)
Unspecified (Q899)	2 (3.6)
Total	55 (100)

Table 3 shows the total number of deaths due to CAs in LB during the period. The majority were early neonatal (n=63; 54.8%), i.e., less than 7 days old. CHD was also the most frequent category in 34.8% of CA deaths (60.0% of these were unspecified CHD). NTD was the second most frequent category in 15.5% of total deaths. Musculoskeletal defects and oral clefts were reported mainly on the Sinasc (Table 1). On the other hand, CHD notifications were mainly recorded in the SIM (Tables 2 and 3).

**Table 3.** Congenital anomalies as a cause of infant death (SIM/Sinasc joint analysis).

Congenital anomalies	n (%)	Mortality rate*	Early neonatal n (%)	Late neonatal n (%)	Post-neonatal n (%)
Circulatory system	40 (34.8)	10.7	17 (27.0)	9 (52.9)	14 (40.0)
Nervous system	18 (15.7)	4.8	15 (23.8)	1 (5.9)	2 (5.7)
Chromosomal disorders	11 (9.6)	2.9	2 (3.2)	3 (17.6)	6 (17.1)
Digestive system	10 (8.7)	3.2	3 (4.8)	0 (0)	7 (20.0)
Multiple	8 (7.0)	2.1	5 (7.9)	2 (11.8)	1 (2.9)
Abdominal wall	7 (6.1)	1.9	5 (7.9)	0 (0)	2 (5.7)
Urinary system	7 (6.1)	1.1	6 (9.5)	1 (5.9)	0 (0)
Respiratory system	6 (5.2)	1.6	5 (7.9)	0 (0)	1 (2.9)
Limb and musculoskeletal defects	3 (2.6)	0.8	3 (4.8)	0 (0)	0 (0)
Cleft lip and palate	1 (0.9)	0.3	1 (1.6)	0 (0)	0 (0)
Unspecified	4 (3.5)	1.1	1 (1.6)	1 (5.9)	2 (5.7)
Total	115 (100)	30.7	63 (100)	17 (100)	35 (100)

*For CA IMR, SIM records were linked to Sinasc, and the ratio between the total number of CA deaths recorded in SIM and the total number of LB in that period, multiplied by 10,000, was calculated.

**Figure 3.**
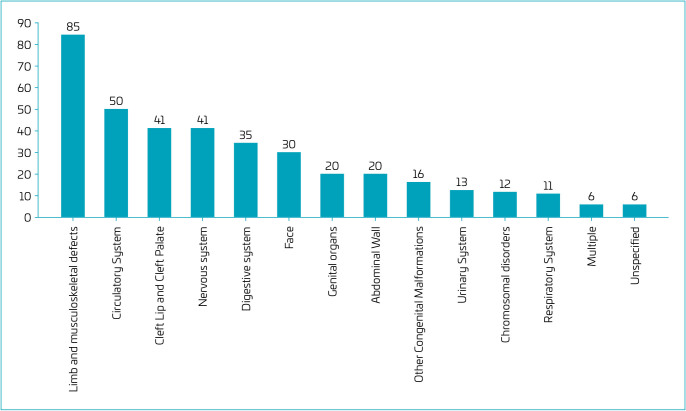
Number of congenital anomalies in the 14^th^ Health Region of Paraná, 2010–2019, after linkage between Sinasc and SIM.

After the linkage between Sinasc and SIM, CHDs became the second most frequent group of CAs (Figure 3). Among the 50 CHDs, 31 (62.0%) were recorded only among the causes of death (Table 2) and not reported in the Sinasc. Of the 115 deaths due to CAs, only 60 (52.2%) had CAs recorded in the Sinasc. Among the deaths due to CAs recorded at birth, 27 (45.0%) were discordant when comparing Sinasc and SIM. Most were recorded in the Sinasc as multiple malformation syndromes (n=13; 21.7%) or unspecified (n=7; 25.9%), but received some specification or diagnosis in the death record.

## DISCUSSION

In northwestern Paraná, CA was the second leading cause of infant mortality between 2010 and 2019, with an IMR due to CAs of 30.7/10,000 LBs, representing 30.6% of all infant deaths. In comparison, between 2001 and 2017, the proportional mortality of children under 1 year of age due to CAs throughout Brazil was 21.7%, while the IMR due to CAs was 24.4/10,000 LBs.^16^ According to data available from DATASUS – Information Technology Department of the Brazilian Public Health Care System (SUS), in the period from 2010 to 2019, 21.6% of infant deaths in Brazil were associated with CAs, with an IMR per CA of 28.0/10,000 LBs.^8^

The higher IMR for CAs observed in our study compared to Brazil in the same period and using the same sources warrants further investigations. On the one hand, it is important to examine whether there are more prevalent or specific risk factors for the occurrence of CAs in the region. On the other hand, regional socioeconomic conditions may influence mortality in cases involving CAs, contributing to the results observed.

By comparison, in Argentina, the mortality rate from CAs is 26.4/10,000 LBs, with a proportional mortality of 28.3%.^17^ Globally, data from 11 countries participating in the European Surveillance of Congenital Anomalies (EUROCAT) show an average IMR from CAs of 11.0/10,000 LBs,^18^ significantly lower than Brazil’s, mostly due to better care of children with CAs.

However, another important factor to be considered is the legislation regarding the termination of pregnancy due to fetal CAs. In countries where this practice is illegal, higher IMRs from CAs are observed. This suggests that, where termination of pregnancy is permitted, there may be an underestimation of the global burden of disease associated with CAs. Furthermore, this situation makes it difficult to assess progress in prevention measures.^18^

The linkage process demonstrated the importance of this strategy for improving CA prevalence estimates and strengthening epidemiological surveillance. This process also reveals weaknesses, failures, and underreporting, especially in the detection and recording of CAs at birth on the Declaration of Live Births.^16^ One of the main reasons for this underreporting is the difficulty in diagnosing complex CAs in the immediate postpartum period, which is when the Declaration of Live Births is usually filled out.^19^

To address this, some strategies have been developed and adopted in cities such as São Paulo.^20^ For example, when a CA is diagnosed after the Declaration of Live Births has been issued, it is possible to record the information and update the Sinasc database. These measures improve the proper recording of CAs, even after birth. Therefore, in addition to training on properly completing the Declaration of Live Births, it is essential to improve the qualifications of health professionals for the early and accurate diagnosis of CAs, with a view to more reliable and detailed records of these conditions.

This improved surveillance supports a better understanding of the impact of CAs on child morbidity and mortality, and informs public health strategies for prevention and care. Integrating different data sources is a key step in achieving more accurate surveillance. Brazil has additional databases, such as hospital admission records and referral systems within SUS, which can complement Sinasc and SIM.

As expected, musculoskeletal anomalies, being usually visible at birth, were the most prevalent both in the Sinasc and SIM.^16^ CHD, on the other hand, rose from seventh to second place after the linkage between Sinasc and SIM, increasing by 25.5%, which is consistent with the findings of Fernandes et al., where it rose from sixth to second place after correcting the prevalence by 17.9%. This demonstrated that data linkage is an effective tool to improve the quality of information on CAs.^16^ CHDs are difficult to identify at birth because they are not visible,^21^ which explains why most of them were not recorded in the Sinasc. The differences between the CA records in Sinasc and SIM are to be expected, showing that many cases are not identified at birth, but are correctly recorded in the death certificate. The integration of different databases is therefore essential.

Nevertheless, there are persistent gaps in the diagnosis and reporting of specific CHD types. The high percentage of “non-specified” types (Q209; Q248; and Q249) makes the surveillance process less effective. It is noteworthy that the majority of CHDs were recorded as unspecified at birth and death, despite the possibility of prenatal diagnosis by ultrasound and fetal echocardiography or detection in the postnatal period with pulse oximetry, a simple and effective screening test.^22^ In Brazil, only 30% of critical CHDs are diagnosed in the fetal period,^23^ and pulse oximetry coverage is unequal between the public health system and private care.^22^ These findings indicate a gap not only in records but also in the early diagnosis of these conditions, which is essential for reducing morbidity and mortality in newborns with CHDs.^21^

NTDs and those related to the nervous system were the third most prevalent group of CAs, mainly anencephaly and spina bifida. The prevalence of NTDs of 5.1/10,000 corroborates the literature, which points to an expected prevalence of 5–6/10,000 LBs in countries with folic acid food fortification.^24^ However, in this study, the same group was the second leading cause of death among CAs, which demonstrates the importance of continuing prevention strategies and early detection.

A study carried out in Colombia,^25^ which analyzed data on fetal and neonatal deaths from CAs, found that CHDs and NTDs were the main anomalies responsible for neonatal mortality, similar to what was observed in this study. Likewise, Fernandes et al. reported that CHDs and NTDs were also the most frequent causes of death due to CAs in Brazil during the period analyzed (2001–2017), with an IMR of 10.0/10,000 LBs for CHDs and 4.4/10,000 LBs for NTDs. In our study (2010–2019), these rates were similar, 10.7 and 4.8/10,000 LBs, respectively.^16^ These two conditions, which can be prevented and treated, are responsible for more than half of all CA-related neonatal deaths.^26^

It is important to observe that some CAs visible at birth, such as anencephaly and gastroschisis, were not recorded in the Sinasc. It is therefore necessary to understand why they were not included in the register. There are various reasons for this, including fear of revealing the diagnosis to the parents or the fact that the form was not filled in by a health professional, as recommended by the Ministry of Health.

In this study, no cases of microcephaly were recorded in either of the information systems (Sinasc or SIM), which could be attributed to the fact that severe microcephaly (which is recorded in Sinasc) has a rare prevalence of 1–3/10,000 LBs. Although the period of our study includes the Zika virus epidemic in Brazil (2015–2017),^27^ a failure to notify cannot be ruled out again.

In addition to the use of folic acid, the prevention of exposure to avoidable risk factors for CA, such as diabetes, obesity, smoking, alcohol consumption, self-medication, and lack of vaccination among women of childbearing age, is fundamental.^28^ Also, the list of priority CAs, organized into eight groups according to ICD-10, is strategic for birth surveillance as it guides Sinasc reporting and highlights the prevention potential of CA at different levels, from early diagnosis to the provision of care, rehabilitation, and the development of public health policies.^15^ This potential is reinforced by continuous epidemiological surveillance of CA-attributable IMR, as well as by expanding access to health services for the population, especially for preconception counseling and prenatal care. These strategies favor early detection and can reduce the IMR associated with CA.^29,30^

Limitations of the present study include the loss of infant deaths at the margins of the study period (e.g., deaths in 2020 of those born in 2019, and deaths in 2010 of those born in 2009), due to the cross-sectional nature of the data. Underreporting is another concern, as CA not recorded at birth (Sinasc) and not notified in SIM may reflect cases never identified or diagnosed later without causing death. These limitations highlight the need to integrate morbidity records to improve the completeness of national databases.

This study highlights the significant impact of CA on infant mortality and emphasizes the urgent need to improve early detection and recording of these conditions, particularly in inland regions of Brazil. Among the anomalies identified, CHDs and NTDs were the most frequent causes of infant deaths associated with CA. These findings reinforce the importance of implementing strategies to enhance CA surveillance and to increase public awareness of preventive measures. Our findings also demonstrate that integrating different data sources can improve the sensitivity and completeness of surveillance systems. Altogether, these actions are crucial to reducing both the prevalence and mortality related to CA, promoting improvements in infant public health in Brazil.

## Data Availability

The database that originated the article is available with the corresponding author.
